# OLDER ADULTS WITHOUT CARE PARTNERS: A SCOPING REVIEW OF CURRENT EVIDENCE

**DOI:** 10.1093/geroni/igae098.0827

**Published:** 2024-12-31

**Authors:** Brittany Jones-Cobb, Chaejeong Lee

**Affiliations:** University of Washington, Seattle, Washington, United States; University of Washington, Seattle, Washington, United States

## Abstract

Demographic changes in rates of living alone, divorce, migration, and having no living partner, spouse, or children are leaving more older adults without the typical “informal” care providers that are the backbone of caregiving in the U.S. These older adults without care partners face a precarious situation given our lack of a robust care structure outside of family/social network members, reductions in publicly funded care, rising costs of privately funded care, and a care workforce shortage. Using the Joanna Briggs Institute guidelines and PRISMA-ScR protocol, we conducted a scoping review of nine databases to synthesize the current evidence regarding this population’s risks and vulnerabilities; adverse consequences; and any interventions that have been developed and tested specifically for this population. Three independent reviewers screened and extracting data, and the first author conducted descriptive and thematic analysis. Precarities facing older adults without care partners included risk for social isolation, impaired self-care, delays and exclusions from medical treatment, and worse medication adherence. Negative outcomes included increased likelihood of hospital and nursing home admission, and death by suicide. No rigorously studied interventions are currently published that specifically target this population. However, some evidence suggested positive benefits from enhancing home-based environmental, formal care, and volunteer supports. Very few of the included articles in this review set out to specifically study older adults without care partners. This highlights our lack of understanding of their distinctive risks and outcomes, and the vital research remaining to develop and test interventions that effectively address this growing population’s unique needs.

